# Scoliosis Corrective Surgery With Continuous Intraoperative Neurophysiological Monitoring (IONM)

**DOI:** 10.7759/cureus.29958

**Published:** 2022-10-05

**Authors:** Faisal R Jahangiri, Rafia H Jahangiri, Hooria Asad, Laila Farooq, Wadana H Khattak

**Affiliations:** 1 Intraoperative Neuromonitoring Program, Labouré College of Healthcare, Milton, USA; 2 Neurology, AINeuroCare Academy, Dallas, USA; 3 Neurophysiology, Global Innervation LLC, Dallas, USA; 4 Department of Neuroscience, School of Behavioral and Brain Sciences, University of Texas at Dallas, Richardson, USA; 5 Surgery, Khyber Medical College, Peshawar, PAK; 6 Surgery, Rehman Medical Institute, Peshawar, PAK

**Keywords:** orthopedic, pediatrics, spine, surgery, scoliosis, neurophysiology, neuromonitoring, ionm

## Abstract

Scoliosis is a spine deformity that presents as Cobb’s angle greater than 10 degrees. Pedicle screw placement can be employed in scoliosis corrective procedures but poses a danger of disrupting the motor and sensory pathways by injuries to the nerves, spinal cord, and vasculature. Occasionally traction weight is applied before the instrumentation for correction. This correction weight may cause spinal cord functional compromise and may result in postoperative paresis or paralysis.

A 10-year-old female patient with Cobb's angle of 120 degrees was scheduled for scoliosis correction surgery. A multimodality intraoperative neurophysiological monitoring (IONM) approach was designed with somatosensory evoked potentials (SSEPs), transcranial electrical motor evoked potentials (TCeMEPs), spontaneous electromyography (s-EMG), triggered electromyography (t-EMG) and train of four (TOF). In this patient, after placing the pedicle screw, TCeMEP changes were immediately identified and reported to the surgeon in the left lower extremity followed by both lower extremities. The surgeon immediately asked the anesthesiologist to remove 25 pounds of traction weight from the head and increase the mean arterial pressure. TCeMEP responses returned to the baselines immediately. Later during the surgery, left arm SSEP changes were also identified, which returned to normal on the repositioning of the arm.

Multimodal IONM has the benefit of monitoring the sensory and motor functions of the spinal cord and nerve function at risk of damage during the procedure. The utilization of IONM in this spinal cord correction surgery helped to detect and timely reverse nerve injuries. We strongly recommend utilizing multimodality IONM during scoliosis correction procedures as a standard of care to minimize postoperative neurological deficits.

## Introduction

Scoliosis is a spine malformation that usually manifests in the early 20 years of life and is defined by Cobb's angle (spinal curve angle) greater than 10 degrees [1,2]. The five primary types are idiopathic, neuromuscular, pathogenic, degenerative, and congenital. The most prevalent kind, idiopathic, accounts for 75 to 80% of all cases [3]. Adolescent idiopathic scoliosis is the most common form, with a prevalence of 0.47 to 5.2% [4] and an equal male-to-female incidence ratio, albeit females are more likely to acquire Cobb's angle larger than 30 degrees [2].

Physical examination, spinal X-ray, computed tomography (CT) scan, and magnetic resonance imaging (MRI) are all used to diagnose scoliosis [5]. Adolescent idiopathic scoliosis can be diagnosed using the forward bend test. Mild scoliosis normally has no serious symptoms other than a backache. However, severe scoliosis (Cobb's angle of 40 degrees or more) can cause discomfort, psychological and social problems, and in rare cases, pulmonary problems [2], but it remains mostly asymptomatic [6].

Scoliosis can be treated with bracing, physiotherapy, and surgery. In addition to the treatment of early onset scoliosis, vertical expandable prosthetic titanium rib (VEPTR) surgery is also used for congenital scoliosis with fused ribs (early onset scoliosis) [7]. Pedicle screws are placed during the instrumentation in scoliosis correction surgery. Still, its proximity to the spinal cord poses a danger of disrupting the motor and sensory pathways [8]. With surgery, there is a danger of spinal cord ischemia, compression, and stretch injury as well as damage to the lumbosacral nerve roots [9].

The vascular supply to the spinal cord follows a pattern from the vertebral vessels to the parenchyma of the white and grey matter throughout the spinal cord. The main supply is via one anterior spinal artery (ASA) and the two posterior spinal arteries (PSA). The ASA provides blood to the anterior two-thirds of the spinal cord, whereas the PSA supplies one-third of the spinal cord. These arteries are further reinforced by segmental branches originating from cervical and lumbar arteries. Since the anterior part of the spinal cord transmits motor signals to different body regions and the posterior part sensory signals [10].

Intra operative neurophysiological monitoring (IONM) is a very reliable method to measure the functional integrity of the central and peripheral nervous system during spinal surgeries [11]. Multimodal IONM also helps assess the integrity of vascular supply by monitoring its corresponding nervous pathways to the spinal cord. It is directed toward detecting possible iatrogenic neurological injuries and providing instant feedback to the surgeon. Hence, it helps to minimize and avoid any postoperative surgical complications. A multimodality IONM approach is recommended, which includes somatosensory evoked potentials (SSEPs), transcranial electrical motor evoked potentials (TCeMEPs), spontaneous and triggered electromyography (s-EMG and t-EMG) and train of four (TOF) [12,13]. Triggered EMG was utilized for pedicle screw stimulation and testing the integrity of the screws.

## Case presentation

Patient selection

**Figure 1:**
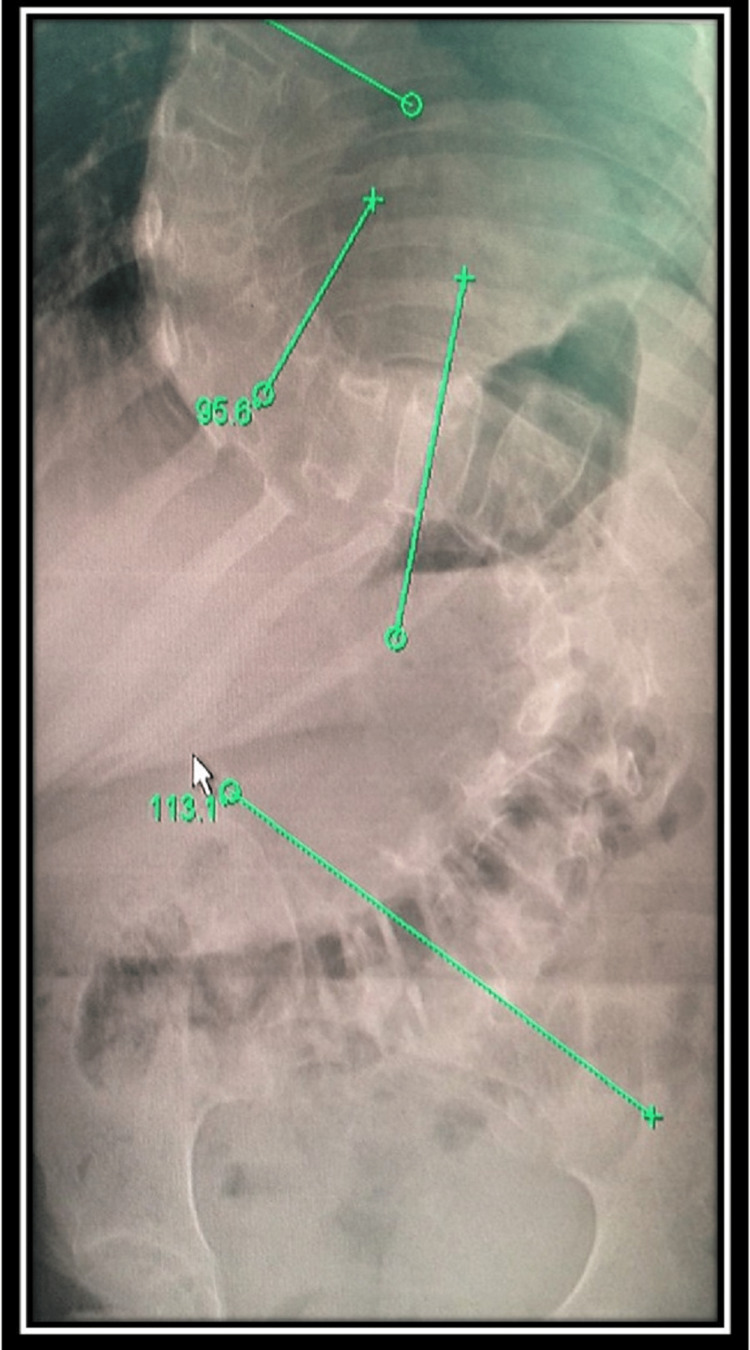
Pre-operative x-ray of the patient showing scoliosis with Cobb’s angle.

A 10-year-old female patient with Cobb’s angle of 120 degrees was scheduled for scoliosis correction surgery using the pedicle screw placement method (Figure 1). The multimodality IONM approach was designed with SSEP, TCeMEP, TOF, and EMG.

Anesthesia

The surgery was performed under total intravenous anaesthesia (TIVA) with propofol and remifentanil. No muscle relaxants were used. The patient was placed in a prone posture with eyes covered and cushioned to avoid cuts, lacerations, trauma, and scratches. However, the Stagnara wake-up exam was explained to the patient before the procedure. A urine catheter, arterial IV-line, temperature measuring equipment, and bite blocks were placed between teeth to prevent harm to the buccal cavity before the surgery.

Multimodal intraoperative neurophysiological monitoring

This surgery utilized a multimodal IONM approach with SSEP, TCeMEP, TOF, and EMG.

Somatosensory evoked potential

SSEP was used intra-operatively to monitor the function of the brain, brainstem, spinal cord, and peripheral nerves as well as an adequate blood flow to the central nervous system. The ulnar and posterior tibial nerves were monitored using surface adhesive stimulation electrodes placed at the wrist and medial malleolus for upper and lower extremity SSEP monitoring. Constant current stimulation of the peripheral nerves of the upper limbs was at 15-25mA and 40-60mA in the lower limbs with a pulse width of 200-300 microseconds used. The low-frequency filters were set to 30 Hz; for high-frequency filters, 500 Hz for cortical and 1500 Hz for subcortical and peripheral recordings. The time display is set to 7.5 milliseconds/division for ulnar nerve SSEP and 12 milliseconds/division for tibial nerve SSEP. The SSEP modality was used to monitor the integrity of the spinal cord's ascending pathways of the dorsal-median column. Real-time feedback regarding the integrity of this pathway was given to the surgeon. The subdermal needle electrodes for SSEP recording were placed according to the international 10-10 system at CP3 (C3'), CP4 (C4'), CPz (Cz'), FPz, and Cv5, Erb’s Point (EPF), and Popliteal Fossa (PF). The alarm criteria to alert the surgeon immediately were set as a 10% or more increase in latency or a 50% decrease in cortical amplitude [14].

Transcranial electrical motor evoked potentials

Corkscrew stimulation electrodes were placed on the scalp according to the international 10-10 system at C1, C2, C3, and C4. TCeMEP stimulation C1-C2 and C3-C4 montages were used for the recordings of the left upper and lower extremities. TCeMEP stimulation C2-C1 and C4-C3 montages were used for the recordings of the right upper and lower extremities. Subdermal needle electrodes are placed for recording in the thenar-hypothenar muscles in the upper extremity and rectus abdominis, adductors, quadriceps, tibialis anterior-gastrocnemius, and abductor hallucis muscles (foot) in the lower extremity. The low-frequency filters were set to 10 Hz and 5 kHz for high-frequency filters. The time display was set to 10 milliseconds/division. Multipulse (five to seven pulses) constant voltage stimulation having a pulse width of 50 microseconds and a stimulation rate of 250-500 Hz was used to elicit TCeMEP. Inter-pulse stimulation interval (ISI) was kept at a range of 2.1-4.1. Stimulation intensity of between 80-350 Volts was used. TCeMEP change was defined as a reduction in amplitude of 70-80% from baseline, a change in waveform morphology from multiphasic to biphasic or monophasic, and an increase in stimulation threshold of 100 Volts or more [12-13,15].

Electromyography

EMG is the measurement of muscle electrical activity. Subdermal needle electrodes were placed in the thenar-hypothenar muscles in the upper extremity and rectus abdominis, adductors, quadriceps, tibialis anterior-gastrocnemius, and abductor hallucis muscles (foot) in the lower extremity. Both s-EMG and t-EMG were recorded for all muscles. Low-frequency filters were set to 10 Hz and 5k Hz high-frequency filters in both s-EMG and t-EMG. The time display rate for s-EMG was set to 300 milliseconds/division and 10 milliseconds/division for t-EMG. Spontaneous EMG (s-EMG) helps detect aberrant activity that could put a nerve root at risk [16].

There are different types of EMG recorded; touching a nerve root with an instrument can cause either a single, biphasic or triphasic spike; these do not indicate any problem or concern [10]. However, a spike train that consists of multiple spikes should be reported to the surgeon immediately as it may be caused by nerve stretching, compression, or heating during surgical procedures. Neurotonic discharge is the most alarming type of recording, usually corresponding to nerve injury and postoperative deficit. Activation of multiple muscle groups can result in complex spike trains, while bumping into a nerve during the procedure can display a burst EMG activity [11,13].

Pedicle screw stimulation with t-EMG assists us in determining the integrity of the pedicle screws during surgical procedures. A constant current was used for t-EMG with a pulse duration set to 0.2 milliseconds and a stimulation frequency of 2.79 Hz.

Train of four

Anesthetized patients must be monitored when IONM with EMG or TCeMEP is utilized for the level of muscle relaxation during the surgery. Four stimulations with a frequency of 2 Hz and a pulse duration of 200 us were applied, with a stimulating current ranging from 0-30 mA. In the lower limb, the tibial nerve was stimulated to record responses from the abductor hallucis and extensor hallucis brevis muscle. The cathode electrode was placed distally at the medial malleolus. The anode electrode is placed approximately 2 cm proximal to the distal electrode. When the paralytic agent is infused, the degree of receptor blockage at the neuromuscular junction assesses the muscle integrity. On stimulation, it appears as twitches (i.e., T1 to T4), with a T4 to T1 ratio indicating fading [17]. 

Case report

A 10-year-old female patient presented as a case of scoliosis with Cobb’s angle of 120 degrees (Figure 1). The surgeon planned a scoliosis correction surgery. Bite blocks were placed after intubation. The multimodality IONM approach was designed with SSEP, TCeMEP, TOF, s-EMG, and t-EMG.

**Figure 2:**
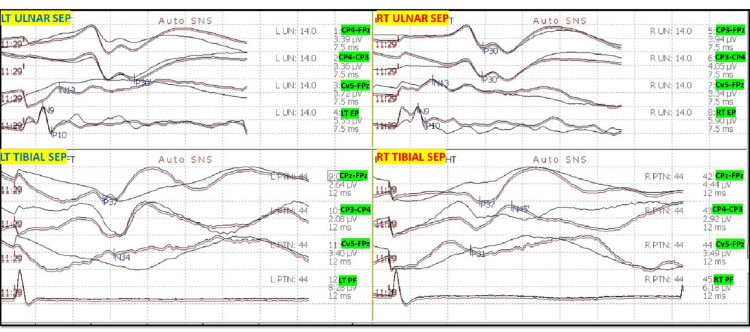
Baseline Somatosensory Evoked Potentials (SSEP) Reliable and reproducible SSEP were recorded at the baselines from ulnar nerves in the upper extremity and posterior tibial nerves in the lower extremities. LT: Left, RT: Right. EP: Erb's Point, PF: Popliteal Fossa.

**Figure 3:**
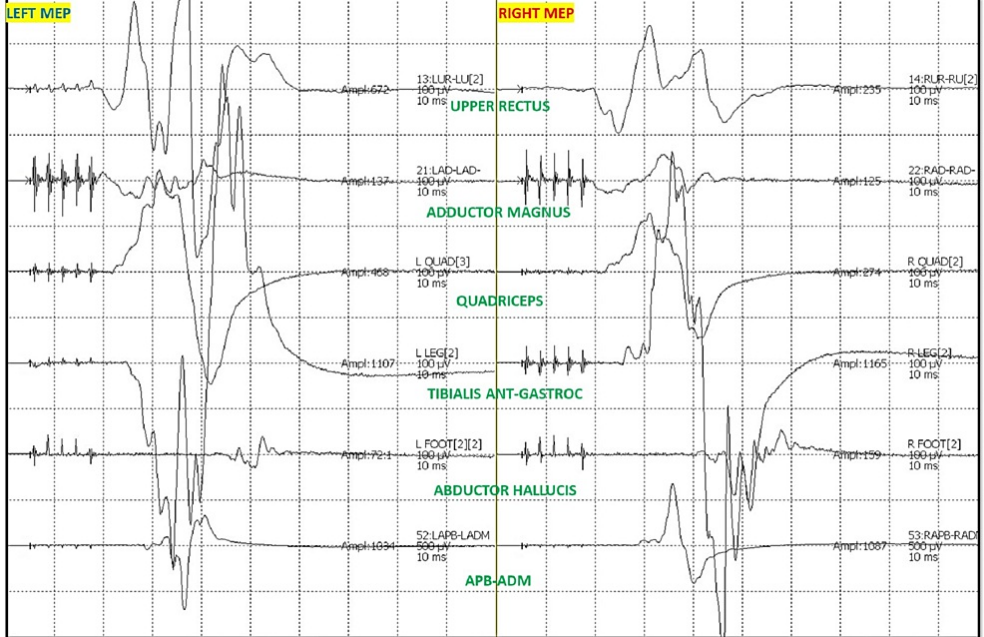
Transcranial electrical Motor Evoked Potentials (TCeMEP) Reliable and reproducible TCeMEP were recorded at the baselines from the upper and lower extremities. ANT: Anterior, APB: Abductor Pollicis Brevis, ADM: Abductor Digitiminimi.

**Figure 4:**
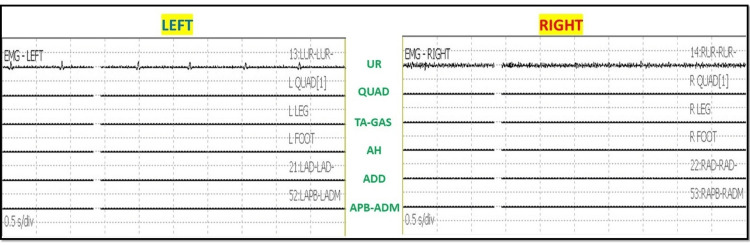
Electromyography (EMG) EMG was recorded at the baselines from the upper extremities (control) and lower extremities. No abnormal EMG signals were present at the baseline. UR: Upper Rectus Abdominis, QUAD: Quadriceps, TA: Tibialis Anterior, GAS: Gastrocnemius, AH: Abductor Hallucis, ADD: Adductor Magnus, Abductor Pollicis Brevis, ADM: Abductor Digiti minimi.

**Figure 5:**
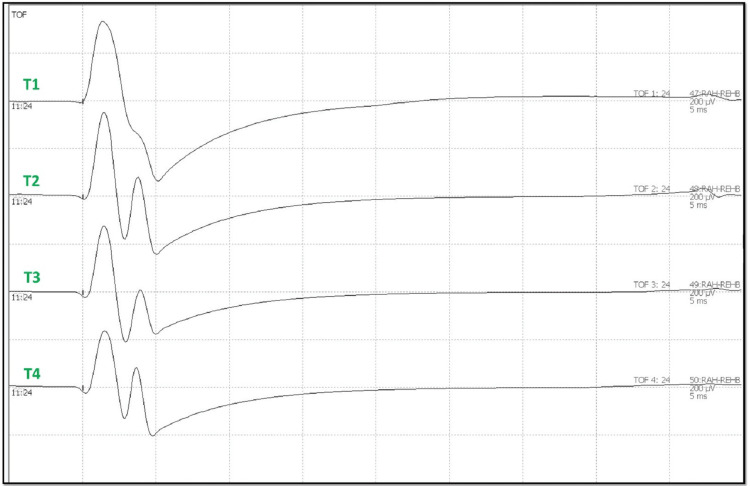
Train of Four (TOF) TOF was recorded at the baselines from the foot muscles (referenced Abductor Hallucis-Extensor Hallucis Brevis) lower extremities. AH: Abductor Hallucis, EHB: Extensor Hallucis Brevis. T1; Twitch 1, T2: Twitch 2, T3: Twitch 3, T4: Twitch 4.

After intubation, electrodes for ulnar SSEP in the upper extremities and posterior tibial nerve SSEP in the lower extremities were placed. The thenar and hypothenar muscles in the upper extremity and rectus abdominis, adductors, quadriceps, tibialis anterior, gastrocnemius, abductor hallucis, and extensor hallucis brevis muscles (foot) in the lower extremity all had electrodes placed for EMG and TCeMEP. Post-induction and post-traction weight placement, upper ulnar nerve SSEP, and lower posterior tibial nerve SSEP were present and reproducible (Figure 2). TCeMEP responses were present in all limbs at baseline (Figure 3). Baseline EMGs were recorded in all muscles (Figure 4), with 4/4 twitches on TOF (Figure 5). The surgeon was informed of these responses.

**Figure 6:**
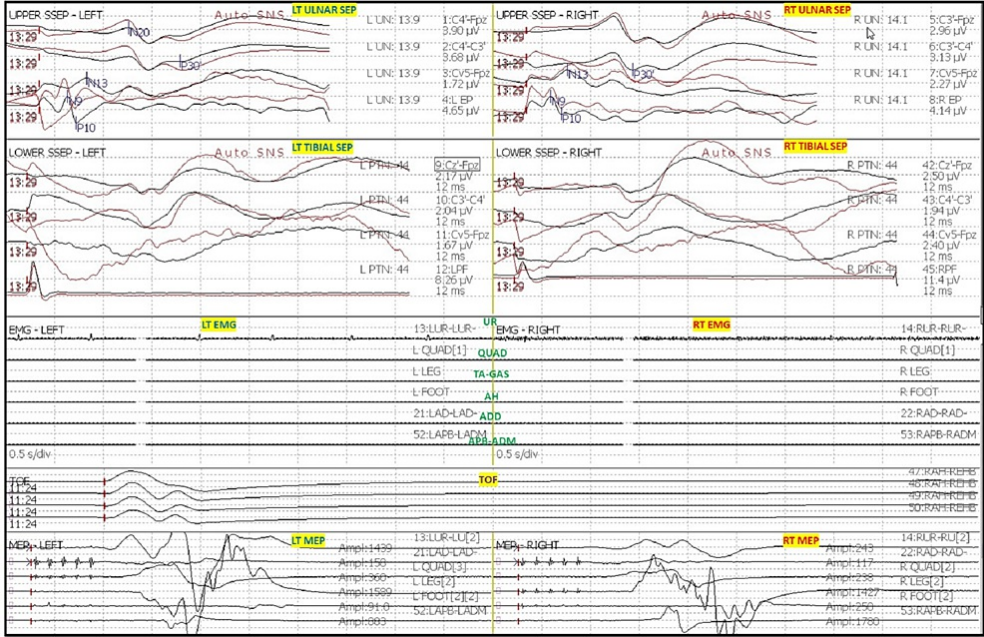
Multimodality Intraoperative Neurophysiological Monitoring (IONM) Baseline IONM data with bilateral upper and lower extremity somatosensory evoked potentials (SSEP), transcranial electrical motor evoked potentials (TCeMEP), and electromyography (EMG). Train of Four (TOF) recorded from foot muscles. LT: Left, RT: Right.

**Figure 7:**
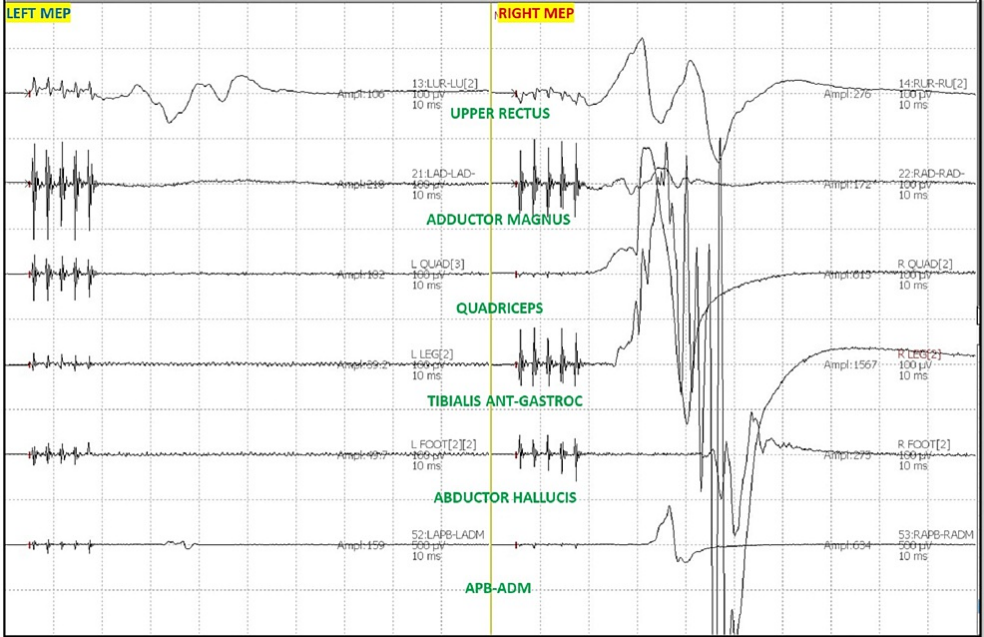
Left Transcranial electrical Motor Evoked Potentials (TCeMEP) changes Changes in the left lower limb TCeMEP data during the surgery. ANT: Anterior, APB: Abductor Pollicis Brevis, ADM: Abductor Digitiminimi.

**Figure 8:**
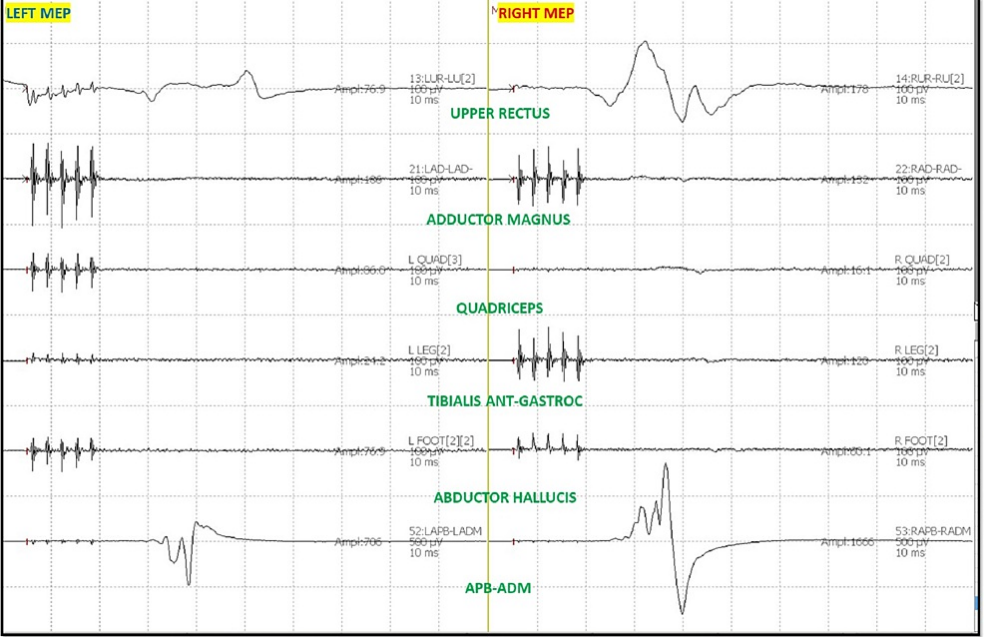
Bilateral Transcranial electrical Motor Evoked Potentials (TCeMEP) changes Changes in the bilateral lower limb TCeMEP data during the surgery. ANT: Anterior, APB: Abductor Pollicis Brevis, ADM: Abductor Digiti minimi.

**Figure 9:**
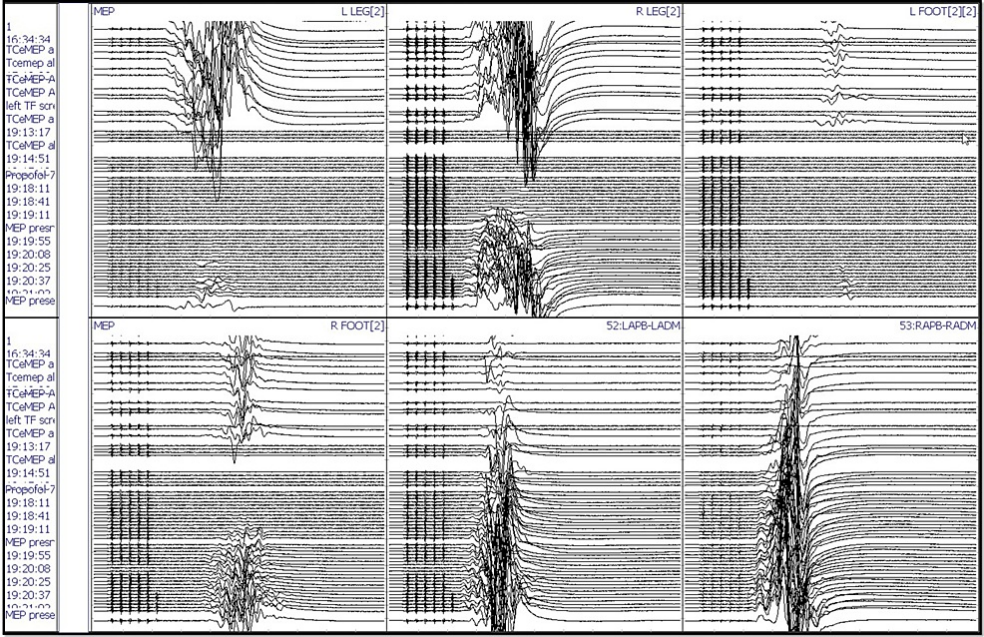
Closing Transcranial electrical Motor Evoked Potentials (TCeMEP) Stack Changes and recovery in the bilateral lower limb TCeMEP data during the surgery. L: Left, R; Right, Leg: Tibialis Anterior-Gastrocnemius, Foot: Abductor Hallucis, APB-ADM: Abductor Pollicis Brevis-Abductor Digitiminimi.

**Figure 10:**
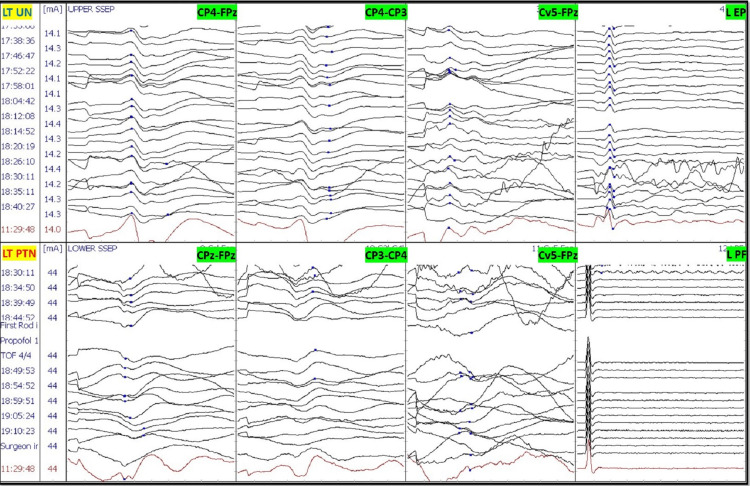
Left Somatosensory Evoked Potentials (SSEP) Stack The closing left SSEP data stack with no changes during the procedure. LT: Left, RT: Right, UN: Ulnar Nerve, PTN: Posterior Tibial Nerve, EP: Erb's Point, PF: Popliteal Fossa.

**Figure 11:**
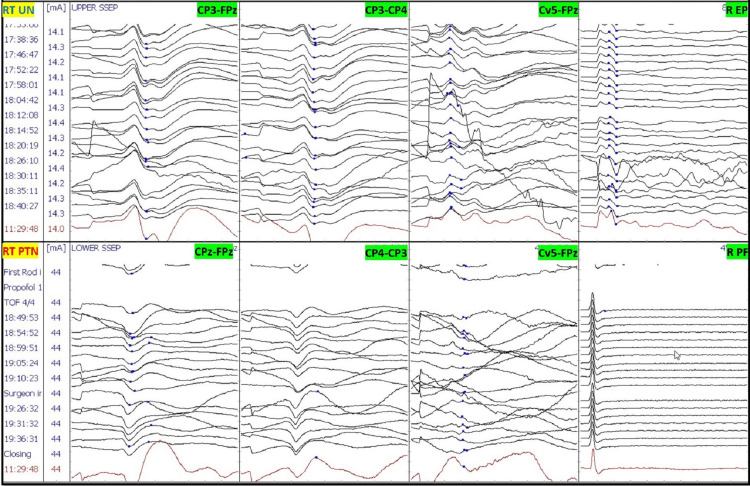
Right Somatosensory Evoked Potentials (SSEP) Stack The closing right SSEP data stack with no changes during the procedure. LT: Left, RT: Right, UN: Ulnar Nerve, PTN: Posterior Tibial Nerve, EP: Erb's Point, PF: Popliteal Fossa.

The patient was placed in a prone position. A multimodality monitoring was performed during the procedure (Figure 6). After exposure, the pedicle screws were placed. The pedicle screw placement was tested with triggered EMG after placement. At 14:24, after the placement of the left L2 pedicle screw, the surgeon was immediately alerted of a drop in the amplitude of the left lower TCeMEP responses (Figure 7). The left L2 screw was immediately removed, but there was no improvement. At 14:28, 25 pounds of traction weight were removed. A minute later, at 14:29, TCeMEP responses recovered to baseline. The surgeon was alerted of the left arm positioning changes in ulnar SSEP at 16:38. The left arm was repositioned, and the left ulnar SSEP was restored to baseline. All pedicle screws showed above the 15 mA acceptable threshold when stimulated with t-EMG. The surgeon was informed about the loss of TCeMEP and a considerable decrease in lower SSEP bilaterally at 19:14, 30 minutes after placing both rods and the correction (Figure 8). The mean arterial pressure (MAP) was recorded at 52 mmHg, and the surgery was paused. MAP was increased to 83 mmHg, which improved TCeMEP and SSEP responses at 19:21, and the surgery was resumed. At 19:47, the surgeon was again informed of amplitude reduction in left lower MEP responses. MAP was 67 mmHg at that time and was increased to 80 mmHg. TCeMEP returned close to the baseline responses (Figure 9). The SSEP responses remained stable bilaterally at closing in the upper and lower extremities (Figure 10, 11]. The surgeon was informed of the closing signals.

Postoperative notes

After completion of the surgery, the patient was extubated. Postoperatively, the patient moved all four extremities and showed no sensory or motor neurological deficits. At three, six and 12 months post-op, the patient showed no neurological deficits.

## Discussion

Scoliosis is a type of spine deformity that, if left untreated, can cause postural, social issues, and breathing problems, as well as life-threatening conditions. Orthopedic surgeons can treat scoliosis with various techniques, with pedicle screw placement being one of them. With the increasing use of pedicle screw placement as a scoliosis correction treatment, various surgical complications have arisen, the most prevalent and severe of which are ischemic and stretch injuries to the spinal cord and muscle paralysis due to nerve and spinal cord injury. These complications can result from improperly placed pedicle screws which have a rate of up to 15.7% [15]. Neurophysiologists employ multimodality intraoperative neurophysiological monitoring techniques during surgical procedures to detect changes in intraoperative neuromonitoring data and alert the surgeon immediately during surgery.

Multimodal IONM has the benefit of collectively monitoring the sensory and motor functions of the spinal cord and nerve function monitoring of the nerves at risk for damage during the procedure. SSEP helps to monitor that the sensorineural pathways of the dorsal part of the spinal cord are intact. They detect nerve ischemia and stretch injuries due to traction as well. In this surgery, SSEP showed a left arm positioning effect which was corrected by repositioning the arm.

TCeMEP helps monitor the functional integrity of the descending corticospinal tracts of the spinal cord. In this surgery, a change in TCeMEP response helped detect possible spinal cord and lumbosacral nerve traction injuries and was reversed immediately. The correction was done by removing some traction weight, and anesthesia was adjusted to increase MAP and bring it back to baseline. In both clinical and research settings, the TOF stimulation method is frequently employed to assess the level of neuromuscular blockade. 

A study by Thirumala et al. of 477 scoliosis corrective spine surgeries reported the sensitivity and specificity of SSEP as 95% and 99.8% respectively [18]. Another study by Thirumala et al. suggested that patients who underwent idiopathic scoliosis surgery and developed a new neurological defect were 250 times more likely to experience alterations in TCeMEPs than patients who did not [19]. Using SSEPs alone can not detect corticospinal cord changes and can only offer tangential evidence of damage to the motor system. Since TCeMEPs are sensitive to tissue ischemia and could identify possible motor deficits earlier than SSEPs, which need averaging over a longer time, they may be able to identify and help to reverse imminent spinal cord injury more quickly than SSEPs. As is typical of TCeMEPs, the high specificity (0.96) supports the use of TCeMEP monitoring as the standard for motor tract neuromonitoring [19]. 

On stimulation, false-negative responses in pedicle screw placement can result from various reasons, such as using muscle relaxants, current spread, or previous nerve injury. With literature finding pedicle screw stimulation thresholds ranging from 6 mA to > 10 mA for a persistently compressed root, as opposed to 2 mA for a normal nerve root, injured nerve roots will have greater triggering thresholds [20].

## Conclusions

Our findings show that intraoperative multimodality IONM during scoliosis correction surgery can help detect iatrogenic neurological impairment early on. As a result, a quick response with suitable intraoperative corrective measures can be used to reduce the severity of the injury and minimize the duration of nerve and spinal cord ischemia and compression. Furthermore, stable multimodal IONM recordings encourage surgeons to proceed with scoliosis corrective procedures. During the surgery, our patient had a deficit of lower TCeMEP responses and positioning changes which were timely detected and reversed due to immediate intervention and continuous neuromonitoring, averting a possible neurological injury. We strongly recommend utilizing multimodality IONM during scoliosis correction procedures as a standard of care.
